# Supplementary material for: Orthogeriatric co-management for older patients with a major osteoporotic fracture: Protocol of an observational pre-post study
Source: PLoS One. 2023 Apr 5;18(4):e0283552. doi: 10.1371/journal.pone.0283552 (PMC10075455; doi:10.1371/journal.pone.0283552)
Supplement: S1 File — (DOCX) [file pone.0283552.s002.docx]

**Protocol Full Title observational trial**:

**Geriatrics and traumatology co-managing fragility fractures: effectiveness and success of implementation in a single centre setting**

**Protocol Acronym/short title**:

G-COMAN FRACTURES

**Version and date of final protocol:**

Amendment 2: 22-04-2022

**Sponsor**:

Name: UZ Leuven

Address: Herestraat 49, 3000 Leuven

**Principal Investigator**:

Name: dr. Marian Dejaeger, MD PhD

Address: Dienst Geriatrie – Herestraat 49, 3000 Leuven

Telephone: 016/34.09.32

Email: marian.dejaeger@uzleuven.be

**Sub-investigator**:

Name: prof. dr. Johan Flamaing, MD PhD

Address: Dienst Geriatrie – Herestraat 49, 3000 Leuven

Telephone: 016/342647

Email: johan.flamaing@kuleuven.be

**Research assistant:**

Name: Sigrid Janssens

Address: Dienst Geriatrie – Herestraat 49, 3000 Leuven

Telephone: 016/344245

Email: Sigrid.janssens@uzleuven.be

Signatures

_________________________ ____________________

Principal Investigator Date

Print Name:

_________________________ ____________________ Date

Print Name:

Table of Contents

[1. Study Synopsis 5](#_Toc336345445)

[2. Background and rationale 7](#_Toc336345446)

[3. Trial objectives and Design 8](#_Toc336345447)

[3.1 Trial objectives 8](#_Toc336345448)

[3.2 Primary endpoints 8](#_Toc336345449)

[3.3 Secondary endpoints 9](#_Toc336345450)

[3.4 Trial Design 10](#_Toc336345451)

[3.5 Study diagram 11](#_Toc336345452)

[3.6 Trial Flowchart 12](#_Toc336345453)

[4. Selection and withdrawal of subjects 19](#_Toc336345454)

[4.1 Inclusion criteria 19](#_Toc336345455)

[4.2 Exclusion criteria 19](#_Toc336345456)

[4.3 Expected duration of trial 19](#_Toc336345457)

[5. Trial Procedures 19](#_Toc336345458)

[5.1 By visit 20](#_Toc336345459)

[5.2 Laboratory tests 20](#_Toc336345460)

[5.3 Other investigations 21](#_Toc336345461)

[6. Assessment of efficacy 22](#_Toc336345462)

[7. Assessment of Safety 22](#_Toc336345463)

[7.1 Specification, timing and recording of safety parameters 22](#_Toc336345464)

[7.2 Procedures for recording and reporting adverse events 22](#_Toc336345465)

[7.3 Treatment stopping rules 22](#_Toc336345466)

[8. Statistics 22](#_Toc336345467)

[8.1 Sample size 22](#_Toc336345468)

[8.2 Analysis 22](#_Toc336345469)

[9. Quality assurance 23](#_Toc336345471)

[10. Direct access to source data and documents 23](#_Toc336345472)

[11. Ethics and regulatory approvals 23](#_Toc336345473)

[12. Data Handling 24](#_Toc336345474)

[13. Data Management 24](#_Toc336345475)

[14. Translational research 24](#_Toc336345476)

[15. Publication Policy 25](#_Toc336345477)

[16. Insurance/Indemnity 25](#_Toc336345478)

[17. Financial Aspects 25](#_Toc336345479)

[18. References 25](#_Toc336345480)

# Study Synopsis

| Title of clinical trial |  | Geriatrics and traumatology co-managing fragility fractures: effectiveness and success of implementation in a single centre setting |
| --- | --- | --- |
| Protocol Short Title/Acronym |  | G-COMAN FRACTURES |
| Sponsor name |  | UZ Leuven |
| Principal Investigator |  | Marian Dejaeger |
| Medical condition or disease under investigation |  | Older fragility fracture patients |
| Purpose of clinical trial |  | 1) To evaluate the effectiveness of a nurse-led geriatric-traumato co-management program using outcome indicators  2) To evaluate the feasibility of a nurse-led geriatric-traumato co-management program  2.1 To evaluate the reach and fidelity  2.2 To evaluate implementation targets 2.3 To evaluate implementation determinants  3)To evaluate the process of implementation of a nurse-led geriatric-traumato co-management program  3.1 To evaluate the reach and fidelity  3.2 To evaluate process outcomes |
| Primary objective |  | 1) Effectiveness evaluation: To determine if geriatric co-management is superior in preventing in-hospital complications |
| Secondary objective (s) |  | 1) Effectiveness evaluation: To determine if geriatric co-management is superior on following outcome indicators:  - length of stay  - unplanned readmissions within 30 and 90 days  - mortality  - functional status  - nutritional status  - quality of life  - falls and new fracture rate  - Discharge to a higher care level  - secondary fracture prevention started  - Cost-benefit of G-COMAN program  2) feasibility and process evaluation: To evaluate if geriatric co-management was successful implemented on the traumato ward:  -2.1 & 3.1: reach of the program and fidelity towards the core components of the care model  - 2.2: implementation targets  - 2.3: implementation determinants  -3.2: process outcomes:  * time to start physiotherapy  * time to start dietary advice  * use and duration of physical restraints  * use and duration of indwelling catheters (urinary tract catheter or intravenous catheter)  * Medication reconciliation at admission and discharge  * referral to geriatric day clinic  * referral to fracture liaison services |
| Trial Design |  | pre-post implementation study on the traumatology ward of the University Hospitals Leuven. |
| Endpoints |  | In-hospital complications (primary endpoint)  Length of stay, unplanned readmissions, mortality, functional status, nutritional status, quality of life, falls and new fracture rate, discharge to a higher care level, secondary fracture prevention, cost-benefit (secondary endpoints) |
| Sample Size |  | Pre-post cohort: Al least 108 patients per group  Feasibility study: 15 patients and 30 health care professionals |
| Summary of eligibility criteria |  | Pre-post cohort: Hospitalised patients aged 75 or older with a fragility fracture on the traumatology ward (E456) of the University Hospitals Leuven  Feasibility study:  - Hospitalised patients aged 75 or older with a fragility fracture on the traumatology ward (E456) of the University Hospitals Leuven  - Health care professionals of E456 will be included if they had four or more weeks of ‘full time’ experience with the programme. |
| Maximum duration of treatment of a  Subject |  | Not applicable |
| Version and date of final protocol |  | Amendment 2: 22-04-2022 |
| Version and date of protocol amendments |  |  |

# Background and rationale

Osteoporosis is the most common musculoskeletal disease and predisposes to fractures of hip, spine, proximal humerus, pelvis and wrist. Approximately around 80.000 fragility fractures occurred in Belgium in 2010 – with associated costs estimated at 606 million euros ^1^. Based on demographic forecasts it is predicted that by 2050 the number of hip fractures will have more than doubled from 2015^2^. These fragility fractures are **associated with pain, disability, need for institutionalization and even death** ^3^. The complex needs of the older fracture patient with multimorbidity, polypharmacy and geriatric syndromes require a holistic, multidisciplinary approach ^4^. This is the expertise of geriatricians and geriatric teams.

At the moment there is little cooperation between geriatricians and surgeons in Belgium due to the current care organization with silo financing. Moreover effects of geriatric consultation teams (as installed by Royal Decree in 2007, update in 2014) are quite limited as repeatedly observed in literature, due to their reactive nature and lack of implementation of advice given^5^. Hence, focus in literature has shifted to different forms of collaboration, such as **co-management with proactive care and shared responsibility** between geriatric and non-geriatric team members. This co)management model can have a significant impact on hospital stay, in-hospital complications and mortality^6^. Based on this convincing data the National Health Service (UK) has **incorporated geriatric care into its guidelines** and hospital reimbursement policy (Best Practice Tariff) resulting in a spectacular nationwide reduction in mortality of hip fracture patients^7^.

Some years ago, we successfully introduced a geriatric co-management model on the cardiology department in our hospital (G-COACH) with significant and clinical relevant results; a reduction in functional deterioration by 18%, better functional status at hospital discharge (Katz ADL 8.9 vs 9.5 in usual care), 13% less delirium and significant fewer nosocomial infections^8^.

Combining the successful results of G-COACH and the orthogeriatric care model abroad, we set up a care strategic project (2021-2024) for geriatric-surgical co-management within the University Hospitals Leuven, named **G-COMAN**. G-COMAN aims to optimize geriatric care in elderly surgical patients through a phased implementation of geriatric surgical co-management on the services traumatology, abdominal surgery and vascular surgery. This year (2021) is dedicated to pre-implementation. From 2022 onwards, G-COMAN will start implementing the program on the traumatology ward. Dedicated nurses will perform comprehensive geriatric assessments (CGA) in all patients aged 75 and older on the traumatology ward. By systematically organizing proactive care with collaboration and shared decision making between geriatric and surgical departments, postoperative care outcomes will hopefully improve.

This observational study will evaluate the **effectiveness of geriatric-traumatology** part of the G-COMAN program and will examine **which factors contribute to** a (non)successful **implementation** of the program as we aim to sustainable embed geriatric care aspects in the standard care of surgical services.

# Trial objectives and Design

### 3.1 Trial objectives

The overall aim of this project is 1) to evaluate the effectiveness of a nurse-led geriatric-traumato co-management program using outcome indicators and 2) to evaluate the implementation of a nurse led geriatric-traumato co-management program

### 3.2 Primary endpoints

1) Effectiveness evaluation

- In-hospital complications, including and limited to:

1/ Delirium: on suspicion of delirium the 4AT^9^ will be used to make the diagnosis. Mini-Cog^9^ will be used to assess the cognitive status of the patient

2/ Congestive heart failure: note in the electronic patient record of heart failure

3/ Pneumonia: diagnosis based on imaging or laboratory testing

4/ Deep venous thrombosis or pulmonary embolism: confirmed on imaging

5/ Myocardial infarction: diagnosis stated in the electronic patient record based on clinical image and laboratory testing/ECG findings.

6/ Urinary tract infection based on laboratory testing and needing treatment with antibiotics

### 3.3 Secondary endpoints

1) Effectiveness evaluation

The secondary endpoints include comparison between the two groups on **patient outcome**:

- Length of hospital stay

- quality of life: EQ-5D^10^ (at admission, discharge, 1 month, 3 months, 6 months and 12 months post-surgery)

- functional status: Parker Mobility Score^11^, modified Barthel Index^12^, Lawton and Brody Scale^13^, Katz index (admission, discharge, 1 months, 3 months and 6 months post-surgery).

- hospital readmissions (30 and 90 days after discharge)

- falls (= “an unexpected event in which the patients comes to rest on the ground, floor or lower level”) and new fractures, either by retrospectively checking electronic patient record for any contact after discharge to any hospital or emergency department stating the occurrence of falls or new fractures) and by a questionnaire send out to patient and care givers. (admission, in-hospital, at discharge, at 1, 3, 6 months post-surgery 12 months post-surgery)

- Living situation: defined in 5 categories: living alone at home, living with spouse/partner, living with children, assisted living, living in a facility with 24h care such as nursing home). Details of care provided will be recorded as one of the following categories: 24h care, daily, irregular, no care. (admission, discharge, 1 month, 3 months post-surgery, 12 months after surgery)

- mortality (in-hospital, 1 month post-discharge and 12 months after discharge)

- secondary fracture prevention: documentation of fall risk assessment, medication review of calcium/vitamin D use and osteoporosis treatment, appointment to fracture liaison service (12 months after surgery)

- nutritional status: Mini Nutritional Assessment (MNA) ^14^ (at admission, 1 month, 3 months and 6 months post-surgery)

- cost-benefit analysis:

Costs will be assessed using a model that registers key hospital activities from the electronic patient record (KWS): 'beeldvorming', 'daghospitalisatie', 'dialyse', 'functiemeting', 'hospitalisatie', 'hospitalisatie ITE', 'labo', 'operatiekwartier', 'paramedische staf', 'raadpleging', 'raadpleging functiemeting', 'radiotherapie', 'spoedgevallen'. The model was developed by UHL.

2. Feasibility evaluation (is the intervention successfully implemented?):

2.1:

- the reach of the program (% of all eligible patients that were included in the G-COMAN program, defined as the patients who completed the questionnaire)

- the fidelity towards the core components of the program (% of included patients who received the core components of the intervention). The core components are 1) the screening questionnaire, 2) comprehensive geriatric evaluation, 3) individual care plan and 4) follow-up.

- The fidelity towards the other intervention components:

- Patient received physiotherapy within 24h postoperative
- The patient was evaluated using the food quadrant method by a logistics employee
- Patient received swallowing screening by a nurse
- If there is no indication for an indwelling catheter, the patient is free of an indwelling catheter
- If a post-void residual volume of ≥ 300 ml is observed in a patient, the residual volume is removed using intermittent catheterization before end of shift after detection of symptoms
- If the urinary catheter was removed, the post-void residual volume is monitored using a bladder scan in the next shift
- If a patient has not passed stool for 3 days, the patient is prescribed oral laxatives before day 4 without stool
- If a patient has not passed stool for 5 days, the patient receives an enema before day 6 without stool
- If there is no indication for a physical restraint, the patients is free of a physical restraint
- If a patient is delirious, the DOS scale was followed up during the next shift
- If a patient reports a pain score of 4 or higher (out of 10), pain medication is given unless refused by the patient within 1h of onset of symptoms
- If a patient reports a pain score of 4 or higher (out of 10), the pain is re-evaluated within 1h of onset of symptoms
- If a patient did not take calcium/vitamin D supplements or anti-osteoporotic medication at admission, it was prescribed at discharge
- The patient was referred to a fracture liaison service

2.2: Implementation targets: awareness, knowledge, motivation to change, perceived acceptability and feasibility, and believe in the benefit, value and success of the program

2.3: Implementation determinants: facilitators and barriers of the program

3. Process evaluation (how well was the process of care changed?)

3.1: reach and fidelity will be reevaluated the same way as expressed above.

3.2: process outcomes

- time to start physiotherapy (as first day after surgery with a first registered contact in electronic patient record)

- % of patients receiving dietary advice and time to start dietary advice

- use and duration of physical restraints (as registered in the electronic patient record; 3-points fixation or higher degrees of fixation)

- use and duration of indwelling catheters (urinary tract catheter or intravenous catheter as registered in the electronic patient record)

- Medication reconciliation at admission and discharge (calcium/vitamin D supplements and AOM)

- referral to geriatric day clinic (appointment to E459 in the year following first admission)

- Referral to fracture liaison services (Zoledronate infusion and/or metabolic bone consultation)

### 3.4 Trial Design

single-centre pre-post implementation study on the traumatology ward of the University Hospitals Leuven

**Usual care (pre-cohort):** the control group receives usual care on the traumato ward. This means that the patient is cared for by a surgical resident. The interprofessional team furthermore consists of ward nurses, a physiotherapist, an occupational therapist and a social worker. No tailored geriatric protocols are available to the traumato team, except for those that are available hospital-wide for all patients (e.g. fall prevention, delirium). Geriatric expertise is available upon active request by the traumato team and includes the comprehensive evaluation of the patient by a geriatric nurse (consultation model).

Due to the design of the G-COMAN strategic project and its predefined strict timing schedule (a phased implementation with the need of a rapidly full implementation), the timeframe for inclusion of the pre-cohort is limited. At the end of the pre-cohort, the predefined sample size will not be reached. To obtain sufficient included patients in the pre-cohort, this patient cohort will be supplemented with patients of which retrospectively information will be gathered. This results in a pre-cohort that will be partly prospective and partly retrospective.

**Geriatric co-management intervention, quality-improvement project G-COMAN (post-cohort):** A dedicated traumato nurse (G-COMAN nurse) will systematically perform a frailty screening in all newly admitted older patients on the traumato ward to evaluate if they are to be included in the G-COMAN program. In eligible patients, a comprehensive geriatric assessment (CGA) will be performed by the G-COMAN nurse. Based on identified potential problems, a tailored care plan based on predefined geriatric protocols will be launched. As part of the project a geriatric specialist nurse will provide every weekday training and education of the G-COMAN nurse and other team members on the traumato ward. The implementation of automated care plans and predefined geriatric protocols will be coordinated by a G-COMAN project coordinator, as well as the organisation of focus groups and intervisions to ensure smooth and supported collaboration.

In between pre-cohort and post-cohort, a single intervention group will be recruited as part of a feasibility study, in order to evaluate if the intervention is implemented successfully. If this is not the case, the implementation will be adapted and revaluation of successful implementation will be planned.

Due to the nature of the study design, health professionals and patients cannot be blinded. Blinding of outcome assessors is not considered feasible due to limited resources.

### 3.5 Study diagram

###


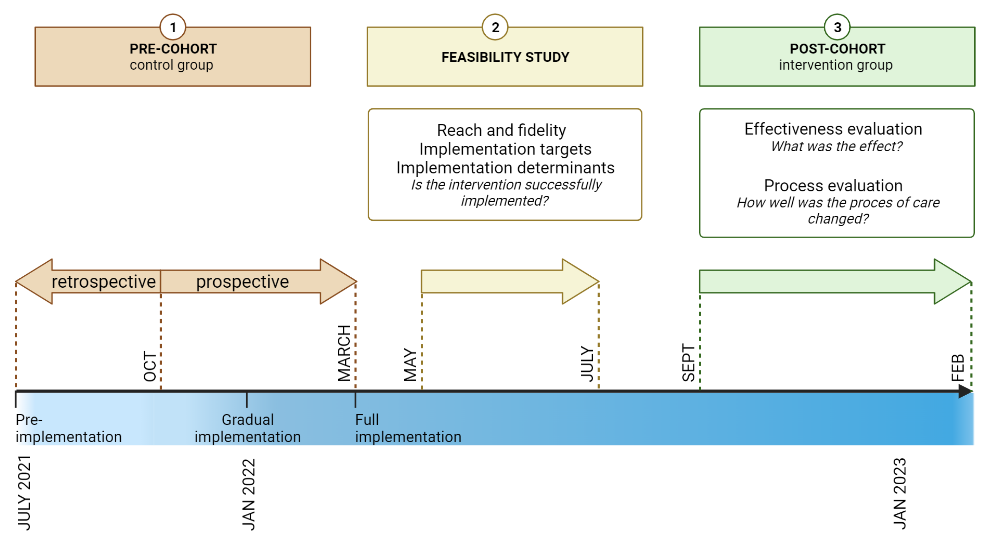


3.6 Trial Flowchart

### *Baseline variables and effectiveness outcomes including time points in the prospective pre- and post-cohort and retrospective pre-cohort.*

| **Variable** | **Instrument** | **Description** | **Score** | **Type of assessment** | **Prospective pre-cohort/post-cohort** | | | | | | **Retrospective pre-cohort** |
| --- | --- | --- | --- | --- | --- | --- | --- | --- | --- | --- | --- |
|  |  |  |  |  | **Admission** | **Discharge** | **1M FU** | **3M FU** | **6M FU** | **12M FU** | **Yes/No** |
| Demographics | N/A | Age, gender |  | Record | X |  |  |  |  |  | Yes |
| Comorbidities | Age-adjusted Charlson Comorbidity Index | Assessment of 16 medical conditions scored based on severity and age | 0-37 | Record | X |  |  |  |  |  | Yes |
| Activities of Daily Living (ADL) | Katz index | Bathing, dressing, toileting, transferring, continence, feeding | 6-18 | Questionnaire | X | X | X | X | X |  | Only baseline via record |
|  | Modified Barthel index | Bowels, bladder, grooming, toilet use, feeding, transfers, mobility, dressing, stairs, bathing | 0-100 | Questionnaire | X | X | X | X | X |  | Only baseline via record |
| Instrumental ADL | Lawton and Brody scale | Telephone use, shopping, food preparation, housekeeping, laundry, mode of transportation, medication use, finances | 0-8 | Questionnaire | X | X | X | X | X |  | Only baseline via record |
| Nutritional status | Mini nutritional assessment | 6 screening questions | 0-14 | Questionnaire | X |  | X | X | X |  | Only baseline via record |
| Mental status | Mini-cog | Three-item word memory and clock-drawing | 0-5 | Test | X | X |  |  |  |  | Only baseline via record |
| Injury and surgery details | N/A | Type of fracture  Type of surgery  Time to surgery  ASA score |  | Record |  | X |  |  |  |  | Yes |
| In-hospital complications | N/A | Delirium  Congestive heart failure  Pneumonia  Deep venous thrombosis  Pulmonary embolism  Myocardial infarction  Urinary tract infection |  | 4AT  Record |  | X |  |  |  |  | Yes, record |
| Length of hospital stay | N/A |  |  | Record |  | X |  |  |  |  | Yes |
|  |  |  |  |  |  |  |  |  |  |  |  |
| Unplanned readmissions | N/A |  |  |  |  |  | X | X |  |  | Yes |
| Mortality | N/A |  |  | Record |  | X | X |  |  | X | Yes |
| Living situation | N/A |  |  | Questionnaire | X | X | X | X |  | X | Only baseline and discharge via record |
| Quality of life | EQ-5D | Mobility, self-care, daily activities, pain, anxiety |  | Questionnaire | X | X | X | X | X | X | No |
| Medication | N/A | Use of calcium/Vitamin D supplements and/or  anti-osteoporotic medication  Polypharmacy (≥ 5 medications) |  | Questionnaire  record | X | X | X | X | X | X | Only baseline and discharge via record |
| Falls and fractures history | N/A | Fall = “an unexpected event in which the patient comes to rest on the ground, floor or lower level” |  | Questionnaire  Record | X | X | X | X | X | X | Only baseline and discharge via record |

M = month, FU = follow-up.

***Process outcomes in the prospective pre- and post-cohort and retrospective pre-cohort.***

| **Variable** | **Instrument** | **Description** | **Score** | **Type of assessment** | **Prospective pre-cohort/post-cohort** | | | | | | **Retrospective pre-cohort** |
| --- | --- | --- | --- | --- | --- | --- | --- | --- | --- | --- | --- |
|  |  |  |  |  | **Admission** | **Discharge** | **1M FU** | **3M FU** | **6M FU** | **12M FU** | **Yes/No** |
| Reach | NA | Only post-cohort: Number of eligible patients included within G-COMAN | NA | NA |  | X |  |  |  |  | Yes |
| Fidelity | NA | Only post-cohort: Number of patients receiving the core components of G-COMAN | NA | NA |  | X |  |  |  |  | Yes |
| Physical therapy | NA | Number of patients receiving physiotherapy  Numbers of days until start physiotherapy | NA | NA |  | X |  |  |  |  | Yes |
| Dietary advice | NA | Number of patients receiving dietary advice  Number of days until start of dietary advice | NA | NA |  | X |  |  |  |  | Yes |
| Physical restraints | NA | Number of patients being restrained  Duration of the use of restraints  Type of restraints used | NA | NA |  | X |  |  |  |  | Yes |
| Indwelling catheters | NA | Number of patients with an indwelling catheter (urinary tract catheter or intravenous catheter)  Duration of catheterization  Reason for catheterization | NA | NA |  | X |  |  |  |  | Yes |
| Medication reconcilliation | NA | Number of patients with calcium/vitamin D supplements and/or AOM | NA | NA |  | X |  |  |  |  | Yes |
| Referral to geriatric outpatient clinic at discharge | NA | Number of patients referred to the falls clinic, memory clinic, follow up | NA | NA |  | X |  |  |  |  | Yes |
| Referral to FLS | NA | Number of patients referred for Zoledronate infusion or metabolic bone consultation | NA | NA |  | X |  |  |  |  | Yes |

M = months, FU = follow-up.

# Selection and withdrawal of subjects

### 4.1 Inclusion criteria

Patients:

- 75 years of age or older

- male or female

- admitted to the traumatology ward (E456) with an osteoporotic fracture

- Proximal femoral fracture
- Proximal humeral fracture
- Fracture of the pelvis / acetabulum
- Fracture of the thoracic/lumbar vertebrae (multiple fractures are allowed)
- Fracture of the wrist

- ability of the patient or assigned representative to understand the content of the patient informed consent form (see **appendix 2** for the pre-post cohort and **appendix 2.1** for the feasibility study).

- signed and dated informed consent.

Health care professionals:

- Working on E456

- Had four or more weeks of ‘full time’ experience with the program.

### 4.2 Exclusion criteria

- patient in palliative care setting not expected to live longer than 3 months.

- multi-trauma injuries except for presence of multiple vertebral fractures

- patient or assigned representative are unable to speak and/or understand Dutch

- periprosthetic fracture

- concomitant joint-infection

- admission from another hospital or hospital unit (reason: no full baseline data on premorbid conditions such as mobility, functional status, … available)

### 4.3 Expected duration of trial

Start Oct 2021 with inclusions of first patients in pre-cohort, till March 2022 with 1 year follow-up. Patients in the feasibility study will be recruited before start of the post-cohort and will start whenever the project team is convinced that the G-COMAN program is implemented as wanted. This will preferably be in the timeframe between May - July 2022. Post- cohort patients will be recruited from Sept 2022 onwards to Sept 2023 with again 1 year follow-up

# Trial Procedures

### 5.1 By visit

### Baseline assessment

All patients who were screened for the inclusion and exclusion criteria will be entered on the ‘Subject screening log’ (see appendix 1). **Demographical data** (gender, age, height and weight (to calculate the Body Mass Index), date of admission and living situation), American Society of Anaesthesiologists (ASA) physical status class^15^, comorbidities (age adjusted charlson comorbidity Index (ACCI) will be all collected by reviewing the patients’ medical record. **Functionality** (Parker Mobility Score, modified Barthel Index, Lawton and Brody Scale, Katz index), **quality of life** (EQ-5D), **fall history and residential status** will be assessed referring to the patient’s pre-injury status. **Mental status**, based on cognition, and **nutritional status** will be assessed with respectively Mini-cog and mini nutritional assessment. Details relative to the **injury** (side affected, fracture location, fracture classification), **surgery** (time to surgery, type of procedure), and total number and intake of relevant **medication** will be documented as well. All variables, except for the quality of life, can be retrospectively collected using the electronic patient record.

### At discharge

The research assistant will monitor the incidence of complications using patient assessment (4AT) and by monitoring the patients record throughout hospitalization, and will assess the outcomes on hospital discharge using patient interview. **Discharge date, medication and mortality** will be evaluated based on the electronic patient record. **Functional status** is measured using the Parker Mobility Score^11^, Barthel Index ^12^, Katz index and Lawton and Brody scale based on reports, if possible from the patient, from next of kin or from nursing staff. **Quality of life** will be evaluated using the EQ-5D questionnaire. **Fall history** will be evaluated using a questionnaire. **Cognition** will be assessed using the Mini-cog test. **Living situation** will be defined within the next 5 categories: living alone at their own home, living with a spouse/partner at their own home, living with children or sibling, living in assisted living facility, living in a facility with 24u care (nursing home, rehabilitation setting,…). Details of care provided will be recorded as one of the following categories: 24h care, daily, irregular, no care. Variables that can be retrospectively collected are as follows: discharge date, medication, mortality, fall history and living situation.

Also **several process outcomes** of the G-COMAN program will be evaluated using registrations in the electronic patient records and by patient interviews: reach of the program (% of eligible patients that were effectively enrolled into the program), fidelity to the program (% of patients included in G-COMAN traumatology program who had a screening or assessment focusing on *delirium, nutritional status, pressure ulcer risk* using a validated tool within 48h of admission to traumatology ward) and specific care process; such as the *time to start physiotherapy*; *time to start dietary advice*; use, *type and duration of fixation;* *use, duration and reason of indwelling catheters (urinary tract catheter or intravenous catheter); medication reconciliation; referral to geriatric day clinic* for follow-up; *and referral to a fracture liaison service (Zoledronate infusion or metabolic bone consultation).*

*Cost-benefit will be calculated by UHL financial division using the UHL cost model based on retrospective data collected at least 3 months post-discharge.*

### 1 month after discharge

Patients or their care givers will receive a letter by post with instructions and an assessment questionnaire for follow-up assessment at 1 month post discharge. In case of no response, the researchers will contact the patient / care giver by telephone to complete the assessment. The following items will be evaluated: **functional status** (Parker Mobility Score, Barthel Index, Lawton and Brody Scale, Katz index), **Quality of Life** (EQ-5D), **residential status**, **nutritional status**, **mortality**, **readmission** and the **presence of new falls and fractures** as well as use of calcium/vitamin D and anti-osteoporosis **medication** in a questionnaire. Only mortality and readmission will be retrospectively collected.

### 3 months after discharge

Patients or their care givers will receive a letter by post with instructions and an assessment questionnaire for follow-up assessment at 3 months post discharge. If case of no response, the researchers will contact the patient / care giver by telephone to complete the assessment. The following items will be evaluated: **functional status** (Parker Mobility Score, Barthel Index, Lawton and Brody Scale, Katz index), **Quality of Life** (EQ-5D), **residential status**, **nutritional status**, **readmission** the **presence of new falls and fractures** as well as use of calcium/vitamin D and anti-osteoporosis **medication** in a questionnaire. Only readmission will be retrospectively collected.

Mean in-hospital costs will be calculated by UHL business manager using the UHL cost model .

### 6 months after discharge

Patients or their care givers will receive a letter by post with instructions and an assessment questionnaire for follow-up assessment at 1 month post discharge. In case of no response, the researchers will contact the patient / care giver by telephone to complete the assessment. The following items will be evaluated: **functional status** (Parker Mobility Score, Barthel Index, Lawton and Brody Scale, Katz index), **Quality of Life** (EQ-5D), **nutritional status**, and the **presence of new falls and fractures** as well as the use of calcium/vitamin D and anti-osteoporosis **medication in a questionnaire**. None of the variables can be retrospectively collected.

### 12 months after admission

**Mortality** will be checked in the electronic patient record (KWS) which is linked the Federale Kruispuntbank and survival status will be double checked with patients/ care givers via questionnaire. **Quality of life** (EQ-5D) and **residential status** will also be checked in the questionnaire. Also the **presence of new falls and fractures** will be asked as well as use of calcium/vitamin D and anti-osteoporosis **medication** in a questionnaire. Only mortality will be retrospectively collected.

### Feasibility study

Before start of recruitment of the post-cohort, a feasibility evaluation in a single intervention group will be performed in order to be sure if the implementation of the intervention is delivered as intended. The feasibility of the intervention will be evaluated using a mixed-methods approach. First, the reach and fidelity (2.1) will be determined with a quantitative evaluation using the electronic patient record. The reach reflects the percentage of eligible patients that was included in the G-COMAN program. Inclusion in the program is defined as having received the questionnaire and a CGA. The number of patients who were actually included will be compared with the total number of eligible patients. The fidelity determines how well the intervention is implemented as defined by the protocol and reflects the percentages of included patients who received the core components of the intervention. Both the core components as the timing will be evaluated. The same baseline characteristics will be collected as in the pre- and post-cohort (variables at admission). Second, a survey will be sent out to all health care professionals working on the traumatology ward measuring the implementation targets (awareness, knowledge, motivation to change, perceived acceptability and feasibility, and believe in the benefit, value and success of the program) (2.2). The survey will consist of questions with each 5 response options: completely agree, agree, neutral, do not agree and completely do not agree (**appendix 3**). Thirdly, focus groups/interviews with all health care professionals will be organised to qualify the implementation barriers, namely the barriers and facilitators of the G-COMAN program (2.3). The expectations and experiences of the multidisciplinary traumatology team will be captured in a focus group/interview before start of the implementation, during the implementation and at the end of the implementation. An interview guide will be drafted to be used during the focus group. One researcher was the moderator and the second researcher observed the discussion and took notes. All interviews will be tape recorded and written out verbatim. Afterwards, the tape records will be deleted.

### 5.2 Laboratory tests

No laboratory tests planned.

### 5.3 Other investigations

No other investigations planned.

# Assessment of efficacy

As this is a pure observational study without any intervention component, therefore no assessment of efficacy of the study concept itself is planned. The implementation of geriatric-surgical co-management on the traumatology ward is linked to the ‘zorgstrategisch project 2021-2024’ in UZL and is not part of this, pre-post observational study.

# Assessment of Safety

### 7.1 Specification, timing and recording of safety parameters

Precautions regarding data protections will be taken as described in section ***12 data handling and management****.* Due to the nature and design of the study (pure observational study) no other safety issues are applicable.

### 7.2 Procedures for recording and reporting adverse events (AE)

Not applicable.

### **7.3 Treatment stopping rules**

Due to the nature and design of the study, there are no stopping rules defined. All treatments are per standard of care and no investigational device or additional medication or intervention is applied during the study.

# Statistics

### 8.1 Sample size

Pre- and post-cohort: Sample size calculation was performed based on difference in the risk of complications. In literature a wide variation in complications rates have been reported ranging from 19.9% to 57% in geriatric co-management and from 55% to 71% in the usual care group ^16-18^. Hence, assuming an absolute reduction by 20%, (alfa:= 0.05, power = 0.8, two sided t-test), equal treatment groups and an expected loss of patients of about 5%; a total of 108 patients are needed per group.

Feasibility study: A total of 15 patients receiving the G-COMAN intervention will be recruited for the feasibility study. Approximately 30 healthcare professionals will be recruited for the focus groups and interviews. The total sample of healthcare professionals will be based on the willingness to participate.

### 8.2 Analysis

Pre- and post-cohort: Categorical data will be expressed as numbers and percentages. Continuous data will be expressed as means with standard deviations. Baseline characteristics will be described and compared between usual care and intervention group to evaluate the baseline equivalence. If equivalent we will test the absolute difference for our primary outcome ‘in-hospital complications’ between the two groups (t-test). If not equivalent, we will test the mean number of in-hospital complications in both groups.

Non-parametric tests will be used to compare baseline characteristics between patients who are losted to follow-up from the control and intervention group. A missing data analysis (multiple imputation) will be performed for baseline characteristics.

In addition, univariable and multivariable regression models will be used whereby the outcome will be the number of in-hospital complications. Following baseline characteristics will be used to control for potential baseline confounding: age, gender, functional status pre-fracture (Katz ADL), physical performance (Parker Mobility score pre-fracture, grip strength), cognitive status (Mini-Cog), multimorbidity (Charlson Comorbidity Index) and nutritional status (Mini Nutritional Assessment).

Secondary analyses will consist of univariable statistical tests (chi^2^ or Fisher’s exact test for categorical variables; t-test or Wilcoxon rank-sum test for continuous variables) to evaluate differences between the two treatment groups. Logistic regression will be used for dichotomous outcomes, survival analyses for time to event variables (with mortality defined as a competing risk for the outcome readmission), and ANCOVA for mean inter-group differences

Longitudinal data will be analyzed by means of mixed effects regression models to estimate differences in mean scores (eg. EQ-5D, modified Barthel Index, Parker Mobility Score, Lawton and Brody Scale) between follow-up and the respective baseline assessment by treatment group. Statistical significance is established at p-value < 0.05.

Enrolled patients who withdraw from the study follow-up for any reason (withdrawal of consent, death, loss of follow-up, …) will be included in the analyses until time at which they withdrew.

Feasibility study: Analysis of the feasibility/process indicators will be reported as frequencies and proportions for categorical data and as mean and standard deviation for continuous data. Qualitative data (focus groups and interviews) will be analysed using a thematic analysis. Two researchers will independently code the data using Word-documents. Integration of quantitative data and qualitative data will occur via embedding by linking data collection and analysis at multiple points.

We are aware of following risk of bias: lack of randomization might induce bias due to the influence of uncontrolled or unbalanced variables. Risk of bias due to death, loss of follow-up or uncompliant patients unable to complete questionnaires is possible. In the latter case, caretakers might help complete the questionnaires if feasible.

# Quality assurance

Appropriate quality assurance measurements will be undertaken:

- Collection and use of data is only possible after formal written consent from the participants (or their representatives).
- Participants will be provided with information related to the study (see appendix 2 ICF) and will have the possibility to opt-out at any moment (see appendix 2 ICF).
- Data will be handled and managed confidential and encoded using REDCAP – all as stated in section 12 below.

# Direct access to source data and documents

The investigator(s) and the institution(s) will permit trial-related monitoring, audits, EC review, and regulatory inspections (where appropriate) by providing direct access to source data and other documents (ie patients’ case sheets, blood test reports, X-ray reports, histology reports etc).

# Ethics and regulatory approvals

| The trial will be conducted in compliance with the principles of the Declaration of Helsinki (current version), the principles of GCP and in accordance with all applicable regulatory requirements. This protocol and related documents will be submitted for review to Ethics Committee and to the Federal Agency for medicinal products for Clinical Trial Authorisation.  The Study can and will be conducted only on the basis of prior informed consent by the Subjects, or their legal representatives, to participate in the Study. The Participating Site shall obtain a signed informed consent form (ICF) for all patients prior to their enrollment and participation in the Study in compliance with all applicable laws, regulations and the approval of the (local) Ethics Committee, if required. The Participating Site shall retain such ICFs in accordance with the requirements of all applicable regulatory agencies and laws.  The Investigator and the Participating Site shall treat all information and data relating to the Study disclosed to Participating Site and/or Investigator in this Study as confidential and shall not disclose such information to any third parties or use such information for any purpose other than the performance of the Study. The collection, processing and disclosure of personal data, such as patient health and medical information is subject to compliance with applicable personal data protection and the processing of personal data (Regulation (EU) 2016/679 also referred as the General Data Protection Regulation ("GDPR") and the Belgian Law of July 30 2018 on the protection of natural persons with regard to the processing of personal data).  Data are **coded**; there continues to be a link between the data and the individual who provided it. The research team is obligated to protect the data from disclosure outside the research according to the terms of the research protocol and the informed consent document. The subject’s name or other identifiers should be stored separately from their research data and replaced with a unique code to create a new identity for the subject. Note that coded data are not anonymous. |
| --- |

# Data Handling and Data Management

Study data will be collected and managed using REDCap®, a secured web application for building and managing electronic surveys and databases. The data will be encoded. Every patient will receive a unique study number and there will be no combination of elements on the data collection forms that allows identification of the individual. Only the principal investigator (MD), the subinvestigator (JF) and the research assistant (SJ) will be able to link the data collected in REDCap® to the patients’ electronic medical record using a subject identification log. The document will be stored separately and in a safe location by the principal investigator (MD) for 10 years, afterwards it will be deleted. The research assistant (x) will introduce the data in REDCap®. The principal investigator (MD) will check correct data collection. Data analysis in the framework of this study will be performed in collaboration with the Leuven Biostatistics and Statistical Bioinformatics Centre. Data collected in this study can be shared with researchers involved in this study upon their request to perform sub analyses. All are affiliated with UZ or KU Leuven. The study does not involve external parties.

# Translational research

No biological material will be collected/shipped/stored/used for the study.

# Publication Policy

Publications will be coordinated by the Investigators. Authorship to publications will be determined in accordance with the requirements published by the International Committee of Medical Journal Editors and in accordance with the requirements of the respective medical journal. The results of the study will be submitted by the core research team as conference abstracts and as publications in professional journals and international peer-reviewed journals.

# Insurance/Indemnity

In accordance with the Belgian Law relating to experiments on human persons dated May 7, 2004, Sponsor shall assume, even without fault, the responsibility of any damages incurred by a Study Patient and linked directly or indirectly to the participation to the Study, and shall provide compensation therefore through its insurance.”

Due to the nature and design of the study (observational study) the risks for patients are considered minimal. Hence, the regular insurance policy of UZ Leuven – KU Leuven is applicable.

# Financial Aspects

Multiple funding options as King Baudoin Foundation (Funds De Lava-Schneider), C2 and FWO predoctoral fellowship will be explored this year (2021) and in the following year.

Meanwhile the PhD student will be employed by the Department of Public Health and Primary Care, Division Gerontology and Geriatrics, KU Leuven.

# References

1. Svedbom A, Hernlund E, Ivergard M, et al. Osteoporosis in the European Union: a compendium of country-specific reports. *Arch Osteoporos.* 2013;8:137.

2. Concin H, Brozek W, Benedetto KP, et al. Hip fracture incidence 2003-2013 and projected cases until 2050 in Austria: a population-based study. *Int J Public Health.* 2016;61(9):1021-1030.

3. Strom O, Borgstrom F, Kanis JA, et al. Osteoporosis: burden, health care provision and opportunities in the EU: a report prepared in collaboration with the International Osteoporosis Foundation (IOF) and the European Federation of Pharmaceutical Industry Associations (EFPIA). *Arch Osteoporos.* 2011;6:59-155.

4. Dyer SM, Crotty M, Fairhall N, et al. A critical review of the long-term disability outcomes following hip fracture. *BMC Geriatr.* 2016;16:158.

5. Deschodt M, Flamaing J, Haentjens P, Boonen S, Milisen K. Impact of geriatric consultation teams on clinical outcome in acute hospitals: a systematic review and meta-analysis. *BMC Med.* 2013;11:48.

6. Patel JN, Klein DS, Sreekumar S, Liporace FA, Yoon RS. Outcomes in Multidisciplinary Team-based Approach in Geriatric Hip Fracture Care: A Systematic Review. *J Am Acad Orthop Surg.* 2020;28(3):128-133.

7. Wilson H. Orthogeriatrics in Hip Fracture. *Open Orthop J.* 2017;11:1181-1189.

8. Van Grootven B, Jeuris A, Jonckers M, et al. Predicting hospitalisation-associated functional decline in older patients admitted to a cardiac care unit with cardiovascular disease: a prospective cohort study. *BMC Geriatr.* 2020;20(1):112.

9. McCarten JR, Anderson P, Kuskowski MA, McPherson SE, Borson S. Screening for cognitive impairment in an elderly veteran population: acceptability and results using different versions of the Mini-Cog. *J Am Geriatr Soc.* 2011;59(2):309-313.

10. Brooks R. EuroQol: the current state of play. *Health Policy.* 1996;37(1):53-72.

11. Parker MJ, Palmer CR. A new mobility score for predicting mortality after hip fracture. *J Bone Joint Surg Br.* 1993;75(5):797-798.

12. Mahoney FI, Barthel DW. Functional Evaluation: The Barthel Index. *Md State Med J.* 1965;14:61-65.

13. Lawton MP, Brody EM. Assessment of older people: self-maintaining and instrumental activities of daily living. *Gerontologist.* 1969;9(3):179-186.

14. Kaiser MJ, Bauer JM, Ramsch C, et al. Validation of the Mini Nutritional Assessment short-form (MNA-SF): a practical tool for identification of nutritional status. *J Nutr Health Aging.* 2009;13(9):782-788.

15. Owens WD, Felts JA, Spitznagel EL, Jr. ASA physical status classifications: a study of consistency of ratings. *Anesthesiology.* 1978;49(4):239-243.

16. Kammerlander C, Roth T, Friedman SM, et al. Ortho-geriatric service--a literature review comparing different models. *Osteoporos Int.* 2010;21(Suppl 4):S637-646.

17. Fisher AA, Davis MW, Rubenach SE, Sivakumaran S, Smith PN, Budge MM. Outcomes for older patients with hip fractures: the impact of orthopedic and geriatric medicine cocare. *J Orthop Trauma.* 2006;20(3):172-178; discussion 179-180.

18. Handoll HH, Cameron ID, Mak JC, Finnegan TP. Multidisciplinary rehabilitation for older people with hip fractures. *Cochrane Database Syst Rev.* 2009(4):CD007125.
