# Supplementary material for: Orthogeriatric co-management for older patients with a major osteoporotic fracture: Protocol of an observational pre-post study
Source: PLoS One. 2023 Apr 5;18(4):e0283552. doi: 10.1371/journal.pone.0283552 (PMC10075455; doi:10.1371/journal.pone.0283552)
Supplement: Attachment — Submitted filename: Response to Reviewers.docx [file pone.0283552.s003.docx]

Dear prof. dr. Blank,

Dear reviewers,

We would like to thank the editor and the reviewers for the time you have invested in reviewing our manuscript. In order to answer all the questions and comments, we have taken the following actions:

- We created a revisions table with three columns: 1) the left column are the reviewers' comments, 2) the middle column are our answers to these comments, and 3) the right column indicates the changes (in **bold**) made in the manuscript.
- The changes in the manuscript were highlighted using track changes.
- Two references were added in the introduction section (reference 10 and 11) in order to clarify a statement that was pointed out by one of the reviewers as you will see in the revisions table.
- A few additional corrections were made by us, these can be found at the end of the revisions table.

We look forward to hear from you at your earliest convenience.

Sincerely,

Sigrid Janssens

| **Reviewers' comments** | **Author’s response** | **Changes made in the manuscript** |
| --- | --- | --- |
| **Reviewer #1** | |  |
| The introduction lays out the context and purpose of the study well, although the authors may consider couching the study's purpose more clearly as a stated, hypothesis that will be tested in introduction or analytical section (since none is formally stated until the discussion line 388, P22). | We agree that the hypothesis should be stated earlier in the manuscript. A hypothesis was added in the introduction. | In the Introduction section, we added the sentence:  **We hypothesize that orthogeriatric co-management for older patients with an osteoporotic fracture will have a beneficial impact on patient outcomes and inpatient costs.** (Line 102-104) |
| The discussion also includes study limitations, which include contamination bias. The discussion might also be expanded to include consideration surrounding portability and generalizability of the proposed intervention to other institutions. The authors may also wish to expand a bit on concerns re: ' history bias ' and what outcomes might be impacted, since the statement beginning on line 443 (p.24) is a bit vague. | Thank you for the suggestions to expand the discussion. However, we feel that those suggestions would fit better in the discussion of the complementary paper about the methods and evaluation of the implementation. That is why we choose to not include these suggestions in this paper.  The statement about history bias was adapted to hopefully make it more clear. | In the Discussion section, we added:  A second limitation of this study is the risk of history bias due to events **(e.g., changing of hospital wide procedures also affecting the study population)** taking place in between the usual care and intervention group that can affect the **study** outcomes. (Line 450-452) |
| The main concern relates to question #5 above relating to usage of 'standard English'. Spelling throughout appears to use British English so if American English usage is preferred, this would need to be addressed throughout the manuscript. | Thank you for pointing this out. Spelling throughout the manuscript was adapted to American English. | Changes were made throughout the entire manuscript. |
| However, there are some grammatical issues that the authors may wish to address. Since this protocol is already in progress, phases that are already completed should be described consistently in past tense. For example beginning on line 16 (p.6), 'prospective inclusion of patients in the usual care group is limited to five months' (should be 'was limited' since the date range occurs in the past and this is otherwise confusing to the reader. Similarly, on p.7 line 122, a feasibility study will be conducted ...' should read 'was conducted' since it has already been done. The sample size estimation should also be stated in past tense (line 137 p.7), and so on. These are just a few examples, but this needs to corrected throughout the manuscript to help the reader to understand what phases are complete and which remain in progress and in fact, since the 'post' period is almost completed, the entire manuscript should be utilizing past tense including protocol and design of measurement frameworks. | Thank you for this remark. We have tried to address your comment, but it becomes very difficult to read the paper changing several times between past and future tense. Therefore, to improve the readability we decided to only write the feasibility study in the past tense, the intervention in the present tense, and the pre-cohort and post-cohort in the future tense. However, we clearly stated the current status of the study in the trial status section to make this more clear for the reader of this protocol. | Changes were made throughout the entire manuscript. |
| Under study participants exclusion criteria, a more precise definition of the what the authors are classifying a 'pathological fracture' would be helpful. For example, I would surmise that osteoporosis was not an exclusion criterion although it would fit the definition a 'pathological fracture'? | Thank you for pointing this out. We added a definition. | In the Study participants section, we added the following:  a pathological fracture **(i.e., fracture caused by cancer)** (Line 141) |
| On line 147 (p. 8) there should be a subheading inserted to labeling that section as 'Feasibility Study' since that phase deserves its own section. Because the discussion of what the feasibility study actually entailed comes much later in the manuscript, the statement re: 'evaluating the feasibility of the intervention' line 150 (p.8) is rather vague, and to help the reader to understand what was actually involved, the authors may consider referring them to Table 2, or ideally, by moving the 'Process indicator variables' text from p.17 to the feasibility section to help the readers' comprehension of the that phase of the study. | Thank you for your comment. However, Line 147 (p. 8) only refers to the sample size calculation of both the effectiveness evaluation and the feasibility evaluation. Thus, inserting a subheading at this point would make it confusing as the methods of the feasibility evaluation comes much later, as you already pointed out. Therefore, a more clear statement about the feasibility study was added in ‘Study design and setting’ section to make the purpose of the feasibility study more clear. | In the Study design and setting section, we added:  After the recruitment of the usual care group and the implementation of the G-COMAN intervention, but prior to the recruitment of the intervention group, a feasibility study will be conducted to evaluate whether **the core components of the G-COMAN intervention were successfully implemented. Once the core components were implemented**, **inclusion of** the intervention group **will be started**. (Line 126 – 131) |
| To assist the authors, a few typos might also be pointed out. On p. 5 line 86, 'that provides in inpatient geriatric consultation' (remove the word 'in': (i.e. that provides inpatient geriatric consultation'. | Thank you for pointing this out. The adjustment was made. | In the introduction section, we removed:  In general, the care for older patients admitted to non-geriatric wards is organised and funded according to the Belgian ‘Care Programme for Geriatric Patients’ (Royal Decree from 2007, updated in 2014) that ~~in~~ provides inpatient geriatric consultation teams. (Line 89) |
| The wording in the discussion might also be smoothed out a bit and presented in more conventional English. For example, consider removing 'on the long term' (not grammatically correct on p. 23 (line 404) and rewording line 403 to read ...immediate phase, to increase likelihood of long-term adoption into daily clinical practice.' | Thank you for the suggestion. We made the adjustment in the discussion section. | In the discussion section, we added:  By continuously focusing on the local context during the whole implementation phase, the intervention is presumed to be successfully implemented in the immediate phase, **holding an increased likelihood of long-term adoption into daily clinical practice** [49]. (Line 407-408) |
| Line 414 (p.23) could be reworded to read: ...multidisciplinary geriatric and surgical team can facilitate diffusion of geriatric expertise to the the care teams. In this manner, ..." | Thank you for this suggestion. We made the adjustment in the discussion section. | In the discussion section, we added:  A transition from a reactive geriatric consultation team model with a one-way approach solely based on input from the geriatric team to a proactive orthogeriatric co-management model with a shared decision making between the multidisciplinary geriatric and surgical team **can facilitate diffusion of geriatric expertise to the care teams. In this manner**, geriatric care can be embedded into the standard of surgical care to reach more geriatric patients. (Line 420-421) |
| To make the sentence on line 446 more grammatically correct, reword as follows: 'We are aware of the limitation introduced by the hybrid prospective/retrospective design of the usual care group.' Again all of the wording in the remainder of that paragraph needs to be converted to past tense, since that phase is complete. | Thank you again for the suggestion. We rephrased this sentence in the discussion section and adapted this part to the past tense. | In the discussion section, we added:  **A last limitation concerns the hybrid prospective/retrospective aspect of the usual care group.** (Line 454-455) |
| On line 455, p. 25, change 'ambition' to 'with high probability of long term sustainability of the intervention'. You may consider splitting that into sentence into 2 two sentences or adding an additional sentence, since you have a 'one sentence paragraph' which is also not grammatically correct, since at least 2 sentences are required to support a paragraph. | The rephasing of this sentence was done. However, we prefer the conclusion to be just one sentence as this sentence represents a short but powerful conclusion of study. We believe that splitting this sentence into 2 sentences would take away the strength of the message. | In the discussion section, we added:  In conclusion, The G-COMAN Fractures study wants to prove the beneficial impact of a newly embedded nurse-led orthogeriatric co-management in improving patient outcomes and costs in a heterogenous population in daily clinical practice with **high probability** of long-term sustainability of the intervention. (Line 463) |
| Finally, the figures were initially confusing since they are not clearly labeled when the manuscript is printed (only visible online) and it was especially confusing because two of the figures are out of order and what is labeled as 'Fig 1' is actually a table. I suspect that this would be adjusted at the time the manuscript is finalized for publication. | ‘Fig 1’ is indeed a table based on the SPIRIT schedule, but is mandatory to submit as figure 1 by PLOS One. | / |
| **Reviewer #2** | | |
| Lines 79-80 – in what diagnoses have the co-management models already been shown to be positive? | The reference used to stated this refers to surgical patients in general (i.e., at the orthopaedic ward, acute surgical unit, and patients admitted for gastrointestinal surgery). This has been more specified. | In the Introduction section, we added:  Geriatric-surgical co-management models **for surgical patients (i.e., orthopedic surgery, gastro-intestinal surgery, and an acute surgery unit)** have shown a beneficial impact on length of stay, mortality, and hospital readmission rates [22]. (Line 80-81) |
| Line 181 – care plans – can you give more details as to what types of care plans will be programmed – physical? Nutritional? | These care plans relate to all four domains (functional, somatic, psychological, and social) as mentioned in the sentence above. To make this more clear, this was added in the next sentence as well. | In the G-COMAN intervention section, we added:  First, all patients aged 75 years and older receive proactive geriatric care interventions that relate to their functional, somatic, psychological, or social status. To support this, various care plans **focusing on those four domains** were automatically programmed in the electronic health record. (Line 189) |
| Line 185 – questionnaire – is this to be filled out by the patient or a caregiver? At what point in the admission will this be done (given that it is about premorbid function and there may be peri-operative/initial delirium)? | Thank you for pointing this out. The questionnaire can be filled in by the patient or by a caregiver and will be sent out at admission to the traumatology ward. It can be filled in during the whole hospitalization and is valid for several months. | In the G-COMAN intervention section, we added:  This questionnaire is sent to the patient **or their caregiver** via the hospital’s mobile application ‘mynexuz.be’ **at admission to the traumatology ward**. (Line 194-195) |
| Lines 281-288 – cost-benefit analysis – will this only consider in-hospital costs/costs associated with the in-patient admission, or will it consider some of the post-admission outcomes (eg hospital readmission, mortality) | The cost-benefit analysis will only consider in-hospital costs. To clarify this, we have added this in this section. | In the Outcome variables section, we added:  The **in-hospital** costs and benefits will be compared between the usual care group and the intervention group. (Line 291-292) |
| **Reviewer #3** | | |
| The justification for this study rests on a potentially inaccurate assumption, though that is only a moderate weakness. The authors assert that, "Osteoporotic fractures most often occur in a frail subgroup of geriatric patients having coexisting multimorbidity, polypharmacy, and geriatric syndromes." The two papers cited in support of this assertion do support the association of frailty with polypharmacy and multimorbidity, but not the assertion that most osteoporotic fractures occur within frail older adults. Throughout the manuscript, it appears that the authors are using "frailty," "older adult," and "osteoporotic" synonymously, which I think is an erroneous approach.  In general, the Introduction makes much about the importance of frailty, but the subsequent methods do not focus exclusively on the frail subset of patients, nor is there any operational definition provided for identifying patients who are genuinely frail. | Thank you for pointing this out. There was indeed no reference supporting the assertion about the prevalence of frailty and major osteoporotic fractures. However, there is literature that supports the assumption about patients with osteoporotic fractures being frail. We added two references to supports this statement.  Since the strength of this study is to include patients with all types of major osteoporotic fractures (thus the daily population on the traumatology ward), the study inclusion criteria did not make a difference between frail and not frail patients. In addition, the intervention itself does not make a difference between patients being frail or not based on the literature supporting a large share of the population with an osteoporotic fracture being frail. That is why we choose to not further elaborate on this. | Two references were added to the following statement in the Introduction section: Osteoporotic fractures most often occur in a frail subgroup of geriatric patients having coexisting multimorbidity, polypharmacy, and geriatric syndromes [8–11]. (Line 56) |
| Further, though the statistics cited about the consequences of a major osteoporotic fracture are congruent with my understanding of the literature, their writing suggests that the proportions of patients experiencing these outcomes are from among only frail patients. Not all patients who have osteoporosis-related fractures meet a definition for frailty, but they are at risk for having outcomes such as functional decline, decreased QoL, and mortality. | We agree with the reviewer that not all patients with an osteoporotic fracture are frail, however, as mentioned above a large percentage of these patients can be categorized as frail. We also agree that patients who do not meet the definition of frailty are at risk for poor outcomes as well. That is why we include these patients as well in the analysis and do not make a difference between frail patients and not frail patients. The risk for patients with frailty having poor outcomes is much greater than for patients who are not frail, therefore, the study will not be underpowered. | / |
| Evaluation of Feasibility - Some additional detail about how the numerous listed process indicators will be used to determine "if the intervention is successfully implemented as intended" seems necessary. It is otherwise difficult to see how these process indicators map to the goal. | To make the goal of the process indicators in the feasibility study more clear, this section was rewritten and an addition has been made to Table 2. | In the Process indicator variables section, we made the following adjustments:  In the feasibility study, the evaluation of the process indicators was used to assess ~~if the intervention is successfully implemented as intended~~ **how well the core components, and several other care processes, of the G-COMAN intervention were implemented as intended in the protocol prior to start of recruitment of the intervention group. The core components of the intervention needed to reach at least 80% of the patients to be feasible.** (Line 296-299)  An addition was also made in Table 2. |
| The target patient population is adults aged 75+ years. A brief justification of this choice would be helpful. | In Belgium, a geriatric patients is defined in the Royal Decree as a patient aged 75 years and over. To make this more clear, we added this in the introduction where a reference was made to this Royal Decree. | In the Introduction section, we added:  In general, the care for **patients aged 75 years and over** admitted to non-geriatric wards is organized and funded according to the Belgian ‘Care Program for Geriatric Patients’ (Royal Decree from 2007, updated in 2014) that provides inpatient geriatric consultation teams [25]. (Line 86) |
| Exclusion criteria include: a) polytrauma - identified how?; b) pathological fracture - cancer related? identified how?; c) concomitant joint infection - identified how? | Thank you for pointing this out. We made this section more clear by adding definitions. | In the Study participants section, we added the following:  a) ~~a polytrauma injury~~ **multiple fractures** (except for presence of multiple vertebral fractures) (Line 139)  b) a pathological fracture **(i.e., fracture caused by cancer)** (Line 141)  c) a concomitant joint-infection **(diagnosed by a physician)** (Line 141) |
| Also excluded are patients transferred from another hospital or ward. So if a person hospitalized for another condition falls while in hospital and breaks a hip, they would not be included? Why? | We made the choice to exclude patients transferred from another hospital or ward after a prolonged admission (> 1 week) prior to the admission to the traumatology ward. In that case, no baseline data would be available. This was adapted in the text. | In the Study participants section, we added the following:  if they are transferred from another hospital or hospital ward **(baseline data not available)** (Line 142) |
| Also excluded are patients with an estimated life expectancy <3 months - how is this determined? | These are patients that are in a palliative setting at admission (thus already known) or become palliative (not fracture-related) during the hospitalization/first 3 months post-fracture. | / |
| Risk of in-hospital complications - does this outcome collectively include all complications of interest? Or individual complications? Is there any intent to analyze complications discretely? It is possible that the intervention may have a differential effect on outcomes and be more effective for reducing some outcomes than for others. | Thank you for this comment. The primary outcome of this study is defined as the proportion of patients having one or more in-hospital complications (with the selection of complications mentioned in the manuscript). This variable will be categorized as ‘yes’ or ‘no’, with patients having one complication or more complications both categorized as ‘yes’. Thus, complications of interest will be collectively analyzed. However, we collect each of these complications separately. This gives us the opportunity to perform sub analyses on the individual complications. | / |
| Outcomes - in-hospital mortality (line 256) - why does this need to come via linkage to the federal database? Would you not otherwise know who has died while in-hospital? | Thank you for pointing this out. The linkage to the federal database is only relevant for the 30-day, 90-day, and 1-year mortality. Therefore, we removed this part of the sentence for the in-hospital mortality. | In the Outcomes section, we removed:  In-hospital mortality. ~~obtained using the electronic health record that is linked to the federal database (‘Kruispuntbank’).~~ (Line 265-266) |
| Outcome - hospital readmissions - are these "all cause" readmissions or fracture-related. If fracture-related, how is it determined that it is fracture-related? | Unplanned hospital readmissions are any cause readmissions. An adjustment was made to make this more clear. | In the Outcomes section, we added:  Unplanned hospital readmissions, defined as being readmitted through the emergency department **for any diagnosis**, will be assessed at 30 and 90 days after discharge. (Line 284) |
| **Additional adaptions that were made by the authors:**   - Trail status was changed: we changed the time frame of inclusion of the intervention group as it stated before that it would end in February 2023. However, to this day, we only included half of the provided sample size. We estimate that the inclusions of the intervention group will end in June 2023. - Study design and setting: the expected duration of the post-cohort was changed to 10 months. In addition, Fig 2 was adapted as well based on this estimation. | | |
